# Development and immunohistochemical characterization of patient-derived xenograft models for muscle invasive bladder cancer

**DOI:** 10.22038/IJBMS.2021.59943.13305

**Published:** 2021-12

**Authors:** Abolfazl Razzaghdoust, Samad Muhammadnejad, Mahmoud Parvin, Bahram Mofid, Masoumeh Zangeneh, Abbas Basiri

**Affiliations:** 1 Urology and Nephrology Research Center, Shahid Beheshti University of Medical Sciences, Tehran, Iran; 2 Gene Therapy Research Center, Digestive Diseases Research Institute, Tehran University of Medical Sciences, Tehran, Iran; 3 Department of Pathology, Labbafinejad Hospital, Shahid Beheshti University of Medical Sciences, Tehran, Iran; 4 Department of Oncology, Shohada-e-Tajrish Medical Center, School of Medicine, Shahid Beheshti University of Medical Sciences, Tehran, Iran; 5 Taleghani Hospital, Shahid Beheshti University of Medical Sciences, Tehran, Iran

**Keywords:** Animal model, Chemotherapy, Muscle-invasive bladder – cancer, Patient-derived xenograft, Targeted therapy

## Abstract

**Objective(s):**

Patient-derived xenograft (PDX) models have become a valuable tool to evaluate chemotherapeutics and investigate personalized cancer treatment options. The role of PDXs in the study of bladder cancer, especially for improvement of novel targeted therapies, continues to expand. In this study, we aimed to establish autochthonous PDX models of muscle-invasive bladder cancer (MIBC) to provide a useful tool to conduct research on personalized therapy.

**Materials and Methods:**

Tumors from MIBC patients undergoing radical cystectomy were subcutaneously transplanted into immunodeficient mice. The tumor size was measured by a caliper twice a week for up to five months. After the first growth in mice, they were serially passaged. Hematoxylin and eosin (H&E) staining and immunohistochemistry (IHC) of 11 markers (Ki67, P63, GATA3, KRT5/6, KRT20, E-cadherin, 34βE12, PD-L1, EGFR, Nectin4, and HER2) were used to evaluate phenotype maintenance of original tumors.

**Results:**

From 10 MIBC patients, two PDX models (P8X20 and P8X26) were successfully established (20% success rate). These models mostly retained primary tumor characteristics including histology, morphology, and molecular nature of the original cancer tissues. IHC analysis showed that the expression level of 7 markers for the model P8X20, and 8 markers for the model P8X26 was exactly similar between the patient tumor and the next generations.

**Conclusion:**

We developed the first autochthonous PDX models of MIBC in Iran. Our data suggested that the established MIBC PDX models reserved mostly histopathological characteristics of primary cancer and could provide a new tool to evaluate novel biomarkers, therapeutic targets, and drug combinations.

## Introduction

Bladder cancer is one of the most common malignancies in men (1). After many years, few changes have occurred in the treatment of muscle-invasive bladder cancer (MIBC) (2). Thus, development of representative MIBC models to evaluate the efficacy of novel targeted therapies and their combinations is required. 

The cancer cell lines do not accurately represent the genetics and heterogeneity of primary tumors, and therefore have limited applicability in translational medicine (3, 4). Since the susceptibility of patient-derived xenograft (PDX) models to anti-cancer drugs is closely correlated with clinical data, these models have proven to be more representative of human patients compared with other xenografts or* in vitro* models (5-7). The PDX models retain the histological and genetic characteristics of the primary tumor and are considered a valuable platform for translational cancer research (8, 9).

Few PDX models for MIBC have been established (10). Since no PDX model for bladder cancer has been developed in Iran up to now, the purpose of this study was to establish and characterize the first autochthonous PDXs for MIBC via implanting the patient tumors into immunodeficient mice. Notably, we used some novel treatment targets (i.e., PD-L1, EGFR, Nectin4, and HER2) and immunophenotype indicators (Ki67, P63, GATA3, KRT5/6, KRT20, E-cadherin, and 34βE12), as immunohistochemical markers to determine the fidelity in characteristics of the established models. Consequently, development and characterization of these MIBC PDX models would provide a useful tool to evaluate the efficacy of potential targeted therapies and their combinations, and thus conduct research on personalized therapy. 

## Materials and Methods

***Animals***

All animals were treated according to guidelines outlined by Institutional Ethical Committee. Female NOD.Cg- Prkdcscid Il2rgtm1Sug/ JicTac (NOG) mice, as the “gold standard” host for PDX models, were used. These mice have a deficiency in the IL-2R g-chain that leads to multiple defects in the innate and adaptive immune system and inhibits NK cell development (11). Mice were provided by Omid Institute for Advanced Biomodels, and kept in the animal lab of Digestive Diseases Research Institute, Tehran University of Medical Sciences (Tehran, Iran), in an individually ventilated cage system under specific pathogen-free conditions on a 12:12 hr light-dark cycle. Autoclaved standard food and water were provided *ad libitum*. Also, room temperature (21 ± 2 °C) and humidity (50 ± 10%) were maintained. 

***Procedures for PDX establishment***

Between April 2019 and October 2019, tumor tissue samples from 10 patients undergoing radical cystectomy for MIBC at Labbafinejad Medical Center and Erfan Hospital were obtained. After removal, tumor tissues were placed into a 50 ml tube containing cold sterile collection medium containing Dulbecco’s modified Eagle medium (DMEM) supplemented with 250 U/ml penicillin, 250 µg/ml streptomycin, and 2.5 µg/ml amphotericin B, and were immediately transferred on ice to the animal laboratory. The subsequent steps and all the *in vivo* procedures were undertaken in a class 2 biological safety cabinet using sterile personal protective equipment. The tumor tissue was transferred to a Petri dish containing cold sterile DMEM. First, all adjacent normal tissues, as well as necrotic tissues, were removed from the tumor. One piece of tumor was fixed in 4% formaldehyde for histopathological examination. The rest of the tumor was gently washed in the medium-containing dish and transferred to a new Petri dish containing the collection medium. The tumor tissue was further divided into small pieces (3 mm^3^). The tumor fragments were placed into a cold sterile 2 ml tube with 1 ml Matrigel (Corning, NY) and incubated for about 10 min before implantation. Subcutaneous pockets were made on flanks in which tumor pieces had been implanted. Multiple tumor fragments were implanted into the flanks of 6–8 week old mice, under balanced anesthesia with 100 mg/kg ketamine and 10 mg/kg xylazine (Alfasan Co, Netherlands), which was defined as F1 mice (12). For each patient specimen, two mice were implanted and monitored for tumor growth for up to five months. 

When the tumor size reached at least 1500 mm^3^, the mouse was euthanized by using 3X anesthetic dose of ketamine/xylazine, and the tumor was passaged for expansion in later serial generations using the same procedure. The tumor size was measured by a caliper twice a week. The tumor volume was calculated as 0.5 × length × width^2^ (13). Tumor growth curves were plotted as tumor volume. We defined the patient-originated specimen as the F0 generation, whereas subsequent generations were numbered consecutively by the number of re-implantations (i.e., F1, F2, and F3).

The study was approved by the ethics committee of Urology and Nephrology Research Center, Shahid Beheshti University of Medical Sciences (IR.SBMU.UNRC.1397.1). The patients provided informed consent.  

***Histopathological examination and immunohisto-chemistry***

Hematoxylin and eosin (H&E) staining and immunohistochemistry (IHC) were used to evaluate phenotype maintenance of the original tumors. 

For H&E staining, fragments of patient samples and PDX tissues were fixed in formalin. The fixed tissues were dehydrated using graded concentrations of ethanol, embedded in paraffin wax, and stained with H&E.  Then, a genitourinary pathologist blinded to the study reviewed each slide to identify whether established PDX models retain the histopathologic characteristics of original patients.  

The IHC protocol was published previously (14). Four novel treatment targets (i.e., PD-L1, EGFR, Nectin4, and HER2), and seven immunophenotype indicators (Ki67, P63, GATA3, KRT5/6, KRT20, E-cadherin, and 34βE12) were used as IHC markers to determine the fidelity in characteristics of the established models. The following primary antibodies were selected for use in this study: Ki67 (BRB040, Zytomed Systems GmbH, Germany), P63 (RMPD086, diagnostic biosystems, California), GATA3 (MAD-000632QD, master diagnostica, Spain), KRT5/6 (PDM123, diagnostic biosystems, California), KRT20 (PDM049, diagnostic biosystems, California), E-cadherin (MAD-000643QD, master diagnostica, Spain), 34βE12 (BMS015, Zytomed Systems GmbH, Germany), PD-L1 (MAD-000740QD, master diagnostica, Spain), EGFR (MAD-000664QD, master diagnostica, Spain), NECTIN4 (HPA010775, Sigma-Aldrich, USA), and HER2 (MAD-000308QD, master diagnostica, Spain). The stained slides were scored by a blinded pathologist using a standard scoring system, based on both intensity and percentage of positive tumor cells (15) (Supplementary Table 1).

***Fluorescence in situ hybridization (FISH)***

The copy number of the HER2 gene, as a potential target in bladder cancer, was assessed by using fluorescence in situ hybridization (FISH) technique. Formalin-fixed paraffin-embedded (FFPE) sections were mounted onto positively charged slides. The slides were incubated for 10 min at 70 °C. Tissue sections were de-paraffinized by xylene two times each for 10 min, followed by rinses in 100%, 100%, 90%, and 70% ethanol, each for 5 min, and a final rinse in water for 4 min. Sections were immersed in a pepsin solution at 37 °C for 15 min, rinsed in wash buffer for 5 min, and in distilled water for 1 min, dehydrated in 70%, 90%, and 100% ethanol for 1 min each, and allowed to dry. Hybridizations were carried out using ZytoLight SPEC HER2/CEN 17 Dual Color Probe (Z-2020-20, ZytoVision GmbH, Germany) in a volume of 10 μl. Slides were coverslipped, sealed with rubber cement, denatured for 10 min at 75 °C, and hybridized overnight at 37 °C. For the post-hybridization and detection process, the rubber cement was carefully removed. Then, the coverslip was removed by submerging in wash buffer at 37 °C for 2 min. The slides were incubated in 70%, 90%, and 100% ethanol, each for 1 min, and allowed to dry in the dark. A volume of 25 μl DAPI/DuraTec-Solution was applied to the specimen to allow visualization of the nuclei. Finally, the slides were coverslipped and incubated in the dark for 15 min. 

## Results

***Establishment of PDX models***

**Figure 1:**
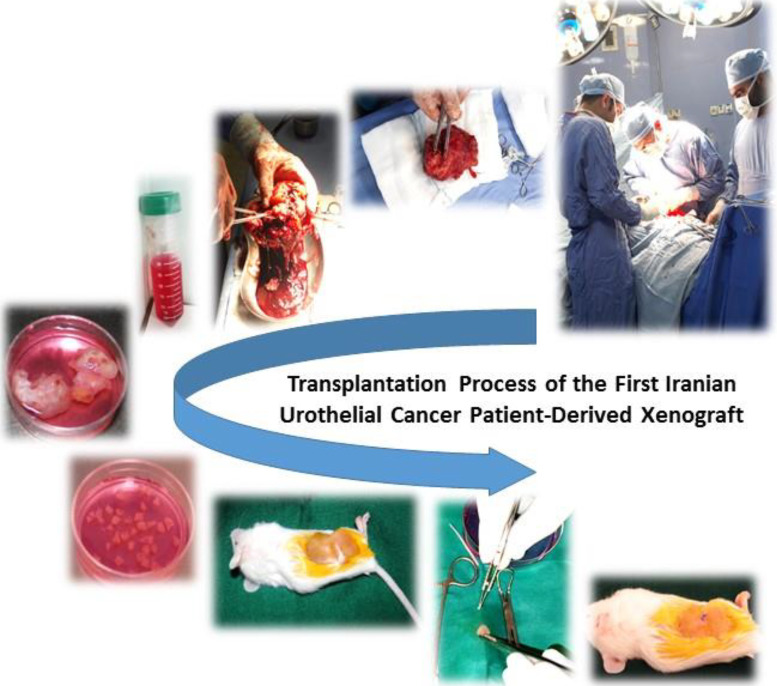
A brief overview of the tumor transplantation process from patients into immunodeficient mice. Tumors from cancer patients (F0) were fragmented and then transplanted into immunodeficient mice (F1) for engraftment

**Figure 2:**
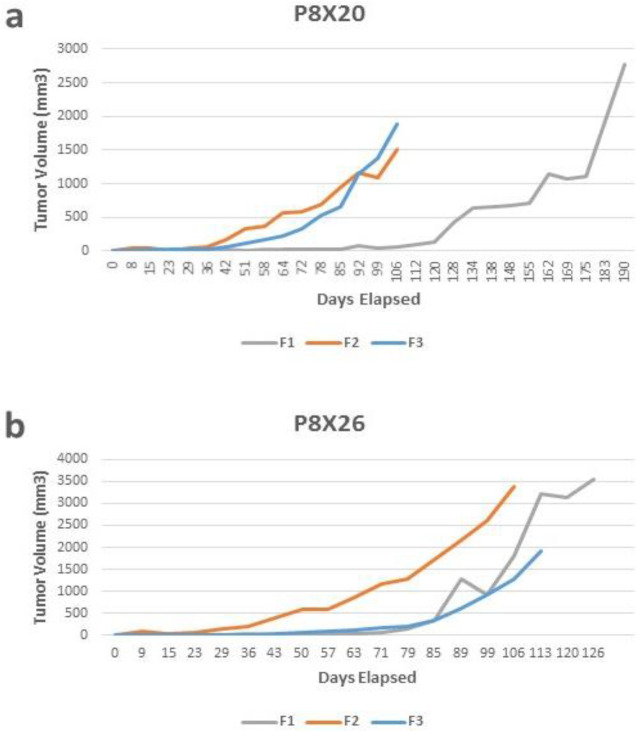
Tumor growth curves of patient-derived xenograft models (a) P8X20 and (b) P8X26. Three passages of xenografts, as represented by F1, F2, and F3, are plotted as tumor volume (mm3) over time

Tumor specimens from 10 patients (F0) were transplanted to 20 NOG mice (F1). A brief overview of this process is shown in Figure 1. The characteristics of tumors and patients from whom the tumor tissues were taken for PDX establishment are shown in Table 1. One patient received prior neoadjuvant chemotherapy. A total of 4 tumors derived from 3 patients grew sufficiently for further transferring. One xenograft had to be excluded after being identified as having post-transplant lymphoproliferative disorder (PTLD). Subsequently, the tumor samples of 2 patients were successfully developed in the mice (20% success rate, defined as the number of xenografts obtained per human tumor sample used). Also, the tumor take rate was 15% (defined as the percentage of mice with xenograft growth divided by the total number of mice implanted). The engraftment rate in the subsequent passages was almost 100% after successful establishment in the first human to mice passage. The consecutive passages (F1, F2, and F3) of two PDX models (P8X20 and P8X26) were successfully expanded. We established a bladder cancer PDX repository, consisting of the original patient tissues and the PDX tissues from each transfer. The PDXs were generated with varying latency times. Figure 2 illustrates the tumor growth curves for three generations (F1–F3) of the established PDXs. For the PDX model P8X20, F2 and F3 exhibited faster growth than that of F1. It took 26 weeks to expand the first passage of the PDX tissue to a volume of 1500 mm^3^ (Figure 2a). Then, it took 15 weeks for the second passage, and 14 weeks for the third passage. After subcutaneously implanting P8X26 tissue, it took 15 weeks to expand the first passage of the PDX tissue to a volume of 1500 mm^3^ (Figure 2b). This time was 12 weeks for the second passage and approximately 15 weeks for the third passage.  

***Histopathologic characterization of PDX models***

The H&E staining and 11 IHC markers (Ki67, P63, GATA3, KRT5/6, KRT20, E-cadherin, 34βE12, PD-L1, EGFR, Nectin4, and HER2) were used to evaluate tumor phenotype maintenance. We found that the PDX models mostly preserved histology, morphology, and molecular nature of original cancer tissues. 

**Figure 3:**
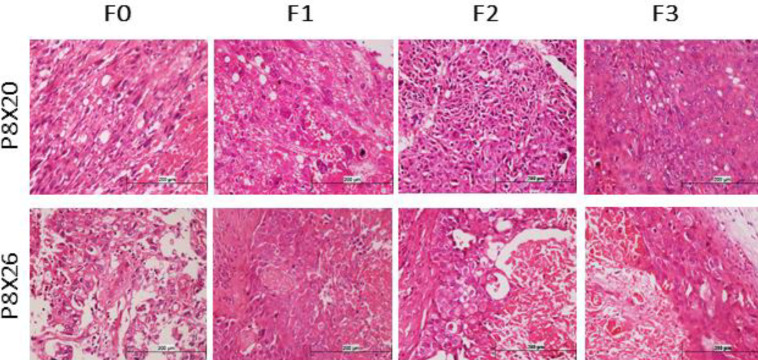
Comparison of histopathologic findings between patients and their matched patient-derived xenografts for the models P8X20 and P8X26. Hematoxylin and eosin stain showed that detailed features of the tumors such as sarcomatoid variant in P8X20, and divergent squamous differentiation in P8X26 were maintained in all generations

H&E staining showed that different generations of the established PDX models preserved the high-grade urothelial carcinoma morphology of the original tumors. Moreover, as shown in Figure 3, detailed features of the tumors such as sarcomatoid variant in P8X20, and divergent squamous differentiation in P8X26 were maintained in all generations. 

**Figure 4:**
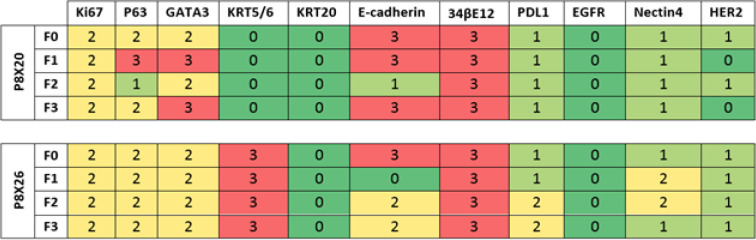
A heat map depicting the level of protein expression for 11 immunohistochemistry markers. Higher protein expression is illustrated in red, and no expression in green. Expression levels of most proteins in primary tumors were retained by different generations of the corresponding patient-derived xenograft

IHC analysis of samples indicated that the expression levels of most proteins in primary tumors were retained by different generations of the corresponding PDXs. A heat map depicting the level of protein expression for 11 IHC markers is illustrated in Figure 4. Interestingly, in the model P8X20, the expression level of 7 markers (i.e., Ki67, KRT5/6, KRT20, 34βE12, PD-L1, EGFR, and Nectin4) was exactly similar between the patient tumor and the next generations. Also, in the model P8X26, the expression of 8 markers (i.e., Ki67, P63, GATA3, KRT5/6, KRT20, 34βE12, EGFR, and HER2) was similar between the original tumor and different generations. Representative images for all 11 IHC markers are shown in Supplementary Figure 1.  

**Figure 5:**
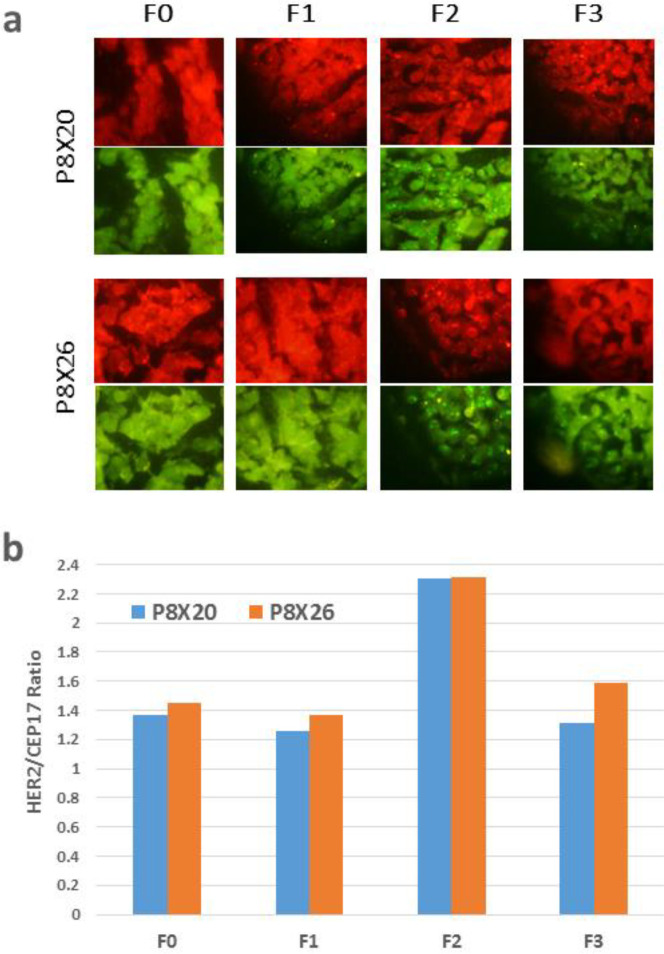
Gene expression analysis in patient tumors and different generations for HER2 gene as a potential target in bladder cancer. (a) Representative images of FISH analysis. The ratios of HER2 probe signal (red) to CEP17 signal (green) represent the amplification status of the HER2 gene (magnification: 100X). (b) A chart for HER2/CEP17 ratio representing the amplification status of HER2 gene in different generations of two patient-derived xenograft models

Analysis of gene expression was performed in patient tumors and different generations for the HER2 gene as a potential target in bladder cancer. The copy number of the HER2 gene and chromosome 17 centromere (CEP17) was assessed by using the FISH technique. The ratios of the HER2 probe signal (red spots) to CEP17 (green spots), shown in Figure 5a, represent the amplification status of the HER2 gene in different generations of two PDX models. As indicated in Figure 5b, the higher amplification of the HER2 gene in the xenograft P8x26 in relation to the xenograft P8X20 was preserved for all generations. Furthermore,  HER2 gene amplification, assessed by FISH, was consistent with HER2 protein expression, assessed by IHC. 

**Table 1:** Characteristics of tumors and patients from whom the tumor tissues have been taken

**Patient No.**	**Sex**	**Age**	**Tumor histology**	**Tumor stage**	**Tumor grade**	**PDX establishment**
1	Male	54	Invasive urothelial carcinoma	T2aNxMx	High	No
2	Male	83	Invasive urothelial carcinoma	T2aNxMx	Low	No
3	Male	62	Invasive urothelial carcinoma	T2bN0Mx	High	No
4	Female	32	Invasive urothelial carcinoma	T3aNxMx	High	No
5	Male	70	Invasive urothelial carcinoma	T2bNxMx	High	No
6	Male	77	Invasive urothelial carcinoma	T1NxMx	High	No
7	Male	51	Invasive urothelial carcinoma	T3aNxMx	High	No
8	Male	59	Invasive urothelial carcinoma	T2bNxMx	High	Yes
9	Male	69	Invasive urothelial carcinoma	T4N0Mx	High	No
10	Male	57	Invasive urothelial carcinoma	T3bN0Mx	High	Yes

**PDX:** patient-derived xenograft

## Discussion

Several* in vitro* and *in vivo* models of cancer have been employed in cancer research. Among these, the PDXs are considered more physiologically relevant to cancers in a clinical setting (16). In our study, the fidelity in histopathological features has been well preserved in the established models. We showed that PDXs not only retained the morphological features, but also the majority of immunohistochemical characteristics of the original tumor. This attractive distinction could provide the possibility to assess the treatment response of candidate drugs before administering to a patient (17). Moreover, it is critical for drug discovery and translatability that a PDX retains the characteristics of the original tumor (9). In our study, some novel treatment targets (nectin-4, EGFR, HER2, and PD-L1) were used as IHC markers to determine the fidelity in immunohistochemical characteristics of the established models. We established the first autochthonous bladder cancer PDX repository in Iran, which could be used in future research. It should be noted that although the success rate from patient to mice passage was fairly low, the engraftment rate in the subsequent passages was almost 100% after successful establishment in the first human to mice passage. Even if the assessment of drug response before clinical administration for a patient seems to be difficult due to the low success rate reported here, our repository could provide screening of multiple therapeutic agents simultaneously to find the most effective drug combination. For example, the combination of T-DM1 as an anti-HER2 Antibody-drug conjugate (ADC) with MIBC chemotherapy regimens is under investigation by using our established PDXs. 

Some previous studies have reported the establishment of bladder cancer PDX. Pan *et al*. established and characterized some PDX models for urothelial carcinoma (18). The authors observed a good correlation between the genetic characteristics of PDX models and original tumors. In another study, Park *et al*. (19) developed 6 PDX models of bladder cancer after implanting 65 primary tumors into the flank of nude mice. In concordance with our results, they revealed the same histologic architecture and degree of differentiation in the primary and xenograft tumors for all six cases. For the majority of IHC markers used in our study, the xenograft models had completely identical IHC scores compared with the original samples. In another study by Jäger *et al*. (17), a total of 7 MIBC tumors were grafted under the renal capsule of female NOD-SCID mice and passaged to establish the bladder cancer PDXs. After excluding one patient, 5 transplantable xenografts were successfully established. Although the establishment of bladder cancer PDXs under the renal capsule is generally associated with a higher success rate, however, establishing this model is technically more complex and requires bioimaging techniques to monitor tumor growth (20). Also, Jäger *et al*. confirmed that the established PDXs retain genetic and histopathologic characteristics of patient tumors. Several other studies reported the outcomes of bladder cancer PDX models, and the reported tumor take rates ranged from 11% to 80% (8). There are differences in details concerning host mouse, site of tumor implantation, tumor histology, tumor sample, whether Matrigel was used or not, and era of PDX model development.

In the PDX model P8X20, F2 and F3 exhibited faster growth than F1, while in P8X26 there was not much difference between the growth rates of different generations. This reflects the fact that the growth rate of subsequent generations can be related to uncontrollable factors such as the tumor characteristics of each patient individually.

This study has several limitations. The small number of patients included in the study can affect the results. The fairly low take rate, long lag period to establish the PDX, and high cost were also crucial limitations as mentioned by other studies (10). Variations in mouse colonies, tumor viability, specimen contamination, surgical technique, and patient factors all likely contribute to the incidence and latency of direct xenograft tumor formation in immunodeficient mice (21).

## Conclusion

We developed and immunohistochemically characterized two MIBC PDXs by directly engrafting tumor tissue samples from patients into immunodeficient mice. The fidelity in histopathological features, in general, has been well preserved in the established PDX models. Since these models have been established for the first time in Iran, thus our PDX repository could be considered a valuable research tool to evaluate the efficacy of potential targeted therapies and their combinations, and thus conduct research on personalized treatment of MIBC patients.

## Authors’ Contributions

AR, SM, and AB designed the experiments; AR, SM, MP, and MZ performed experiments and collected data; AR, SM, BM and AB discussed the results, supervised, directed, and managed the study; AR, SM, MP, BM, MZ, and AB approved the final version of the manuscript to be published.

## Conflicts of Interest

The authors report no conflicts of interest.
